# The Association between *LRRK2* G2385R and Phenotype of Parkinson's Disease in Asian Population: A Meta-Analysis of Comparative Studies

**DOI:** 10.1155/2018/3418306

**Published:** 2018-07-10

**Authors:** Wei Di, Zhiyong Zeng, Jingyan Li, Xiaoling Liu, Minzhi Bo, Hua Lv

**Affiliations:** ^1^Department of Neurology, Xiangya Hospital, Central South University, Changsha, Hunan 410008, China; ^2^Department of Neurology, Shaanxi Provincial People's Hospital, Third Affiliated Hospital of Medical College, Xi'an Jiaotong University, Xi'an, Shaanxi 710068, China; ^3^Pediatric Department, The Second People's Hospital of Longgang District, Shenzhen, Guangdong 518112, China; ^4^Xi'an Medical College, Xi'an, Shaanxi 710068, China

## Abstract

Numerous studies have investigated the relationship between the *LRRK2* G2385R variant and clinical characteristics in Parkinson's disease (PD), but the results have been inconsistent. This study investigated whether the *LRRK2* G2385R variant was associated with a unique clinical phenotype of PD in the Asian population, using a meta-analysis. The PubMed, Web of Science, EMBASE, CNKI, and WANFANG databases were searched until September 2017. The strict selection criteria and exclusion criteria were determined, and mean differences (MD) or odds ratios (OR) with 95% confidence intervals (CI) were used to assess the strength of associations. Statistical analyses and graphics were performed using Review Manager 5.3. Sixteen related case-control studies were included in the meta-analysis. The *LRRK2* G2385R carriers significantly more often presented a family history (OR: 1.98; 95% CI: 1.16−3.39; *P*=0.01) and had a longer disease duration (MD = 0.47, 95% CI: 0.01−0.93, *P*=0.04) and a higher MMSE score (MD = 1.02, 95% CI: 0.43–1.62 *P*=0.0007) than *LRRK2* G2385R noncarriers. There were no significant differences in sex distribution, age at onset, initial symptoms, motor symptoms, depression, levodopa-equivalent dose, and related complications between *LRRK2* G2385R-carrier and *LRRK2* G2385R-noncarrier PD patients. Our results suggested that most of the clinical characteristics of PD patients with *LRRK2* G2385R mutations are similar to those of *LRRK2* G2385R noncarriers among Asian PD patients, except for the more common family history, relatively longer disease duration, and higher MMSE scores in the former group.

## 1. Introduction

Parkinson's disease (PD) is the second most common neurodegenerative disorder. PD is defined by the presence of bradykinesia and at least one of the following symptoms: muscular rigidity, rest tremor, or postural instability. It is characterized by the cardinal motor symptoms of resting tremor, rigidity, bradykinesia, and postural instability, and a variety of nonmotor symptoms, such as olfactory dysfunction, psychiatric disorders, autonomic disturbances, and cognitive decline, among others. Although the etiology of PD has not been fully elucidated, it is thought to be the result of the interaction between genetic and environmental factors [1]. A number of candidate genes involved in PD etiology have been identified. Among them, mutations in the leucine-rich repeat kinase 2 gene (*LRRK2*) have been consistently reported to be the most frequent known cause of both sporadic and familial PD [2]. In particular, the G2385R polymorphism in *LRRK2* is an important genetic risk factor for PD in Asian individuals, as evidenced by various independent studies.

In recent years, genotype-phenotype correlation studies have suggested that PD patients with the *LRRK2* G2385R variant may exhibit some unique clinical characteristics, although some results were contradictory. Available studies have found that *LRRK2* G2385R carriers had a higher frequency of family history [3], longer disease duration [4], a lower age at onset [5], a higher proportion of postural instability and gait disorder (PIGD) phenotype [6], and a higher Mini-Mental State Examination (MMSE) score than PD patients who do not carry the *LRRK2* G2385R [6]. Moreover, a higher levodopa-equivalent dose (LED) and a higher proportion of levodopa-induced complications, including motor fluctuations and dyskinesia, were also observed in PD patients with the *LRRK2* G2385R variant in some studies [6, 7]. However, several other findings indicated that neither demographic data nor clinical presentation differed significantly between *LRRK2* G2385R carriers and noncarriers [8–10].

Taken together, there is no consensus about the relationship between *LRRK2* G2385R and clinical manifestations of PD patients. Here, we performed a meta-analysis to investigate whether *LRRK*2 G2385R was associated with the clinical presentation of PD.

## 2. Methods

The literature search strategies, inclusion and exclusion criteria, outcome measurements, and methods of statistical analysis were completed according to the Preferred Reporting Items for Systematic Reviews and Meta-analysis and Meta-analysis of Observational Studies in Epidemiology recommendations for study reporting [11–13]. All analyses were based on previously published studies; thus, ethical approval and patient consent were not required.

### 2.1. Literature Search Strategy

A literature search was performed for publications up to September 2017 without restriction to regions, publication types, or languages. The primary sources were the electronic databases of PubMed, Web of Science, EMBASE, CNKI, and WANFANG. The following terms and their combinations were searched in [All Fields]: Parkinson disease, Parkinson's disease, Parkinson^∗^, LRRK2 G2385R, LRRK2 Gly2385Arg, LRRK2 c.7153G>A. The reference lists of the included studies were screened to find further relevant studies.

### 2.2. Inclusion and Exclusion Criteria

Studies were selected when they met all the following criteria and the following studies were included if they met any of the following criteria: (1) observational studies, such as case-control studies and cohort studies; (2) comparative studies, which had to include an *LRRK2* G2385R-carrier PD group and an *LRRK2* G2385R-noncarrier PD group; (3) studies of association between specific clinical features (motor symptoms and nonmotor symptoms) and the *LRRK2* G2385R mutation; and (4) the diagnosis of PD had to be made according to the United Kingdom Parkinson Disease Society Brain Bank criteria. The following studies were excluded: (1) review articles, case reports, editorials; (2) duplicated reports (when multiple reports studying the same participants were published, the latest or most complete report was included); (3) studies with incomplete data, which included studies that did not compare *LRRK2* G2385R-carrier and *LRRK2* G2385R-noncarrier PD patients; and (4) functional studies, such as animal experiments and cell experiments studies.

### 2.3. Data Extraction and Outcomes of Interest

Data from the included studies were extracted and summarized independently by two authors (Wei Di and Zhi-yong Zeng). Any disagreements were resolved by the senior authors (Jing-yan Li and Hua Lv). The primary outcomes were the comparison of clinical characteristics including motor symptoms and nonmotor symptoms in the two study groups (*LRRK2* G2385R carriers and noncarriers).

### 2.4. Quality Assessment and Statistical Analysis

The quality of selected studies was evaluated using the Newcastle−Ottawa Quality Assessment Scale (NOS) [14], which included three factors: patient selection, comparability of the study groups, and assessment of outcome. A score of 0–9 was allocated to each study. Studies that achieved six or more points were considered to be of high quality. The assessments were conducted by two authors (Wei Di and Zhi-yong Zeng).

All statistical analyses and graphics were generated using Review Manager 5.3 (Cochrane Collaboration, Oxford, England). The weighted mean difference (MD) and odds ratios (OR) were used to compare continuous and dichotomous variables, respectively. All results were reported with 95% confidence intervals (CIs). Statistical heterogeneity between studies was assessed using the chi-square test with significance set at *P* ≤ 0.10, and heterogeneity was quantified using the *I* ^2^ statistic. The random effect model was used if there was heterogeneity between studies; alternatively, the fixed effect model was used [15]. *Z* tests were conducted to assess the association between the *LRRK2* G2385R variant and clinical characteristics. *P* values < 0.05 indicated statistically significant differences. Sensitivity analysis was performed in each comparison. Funnel plot analyses were used to screen for potential publication bias.

## 3. Results

### 3.1. Characteristics of Eligible Studies

**Figure 1:**
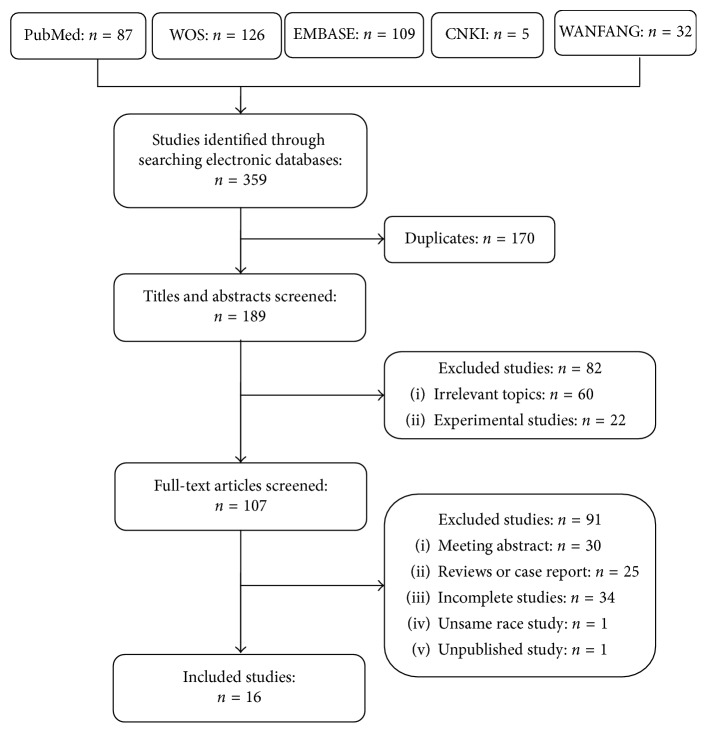
Flow diagram of studies included publication.

A total of 359 studies were identified by searching in PubMed, Web of Science, EMBASE, CNKI, and WANFANG electronic databases. Sixteen eligible studies were included in the final statistical analysis [3–10, 16–23]. The detailed flow chart of study selection and reasons for exclusion are shown in Figure 1. All publications were full-text articles. Agreement between the two reviewers was 97% for study selection and 94% for quality assessment of trials. The detailed characteristics of all included studies are summarized in Table 1. All studies were performed on Asian individuals. The NOS score of all the included studies were no less than 6, indicating that none of the included studies were of low quality.

### 3.2. Meta-Analysis Results

#### 3.2.1. Family History

PD patients with family history are defined as having at least one first- or second-degree relative with a diagnosis of PD. Five studies assessed the relationship between *LRRK2* G2385R status and family history of PD, and the data showed that a family history of PD was significantly more common in the *LRRK2* G2385R carriers than in the *LRRK2* G2385R noncarriers (OR: 1.98; 95% CI: 1.16−3.39; *P*=0.01) (Supplementary Figure 1).

#### 3.2.2. Sex Distribution

Twelve studies included a sex distribution analysis between *LRRK2* G2385R-carrier PD group and *LRRK2* G2385R-noncarrier PD group. There was no significant association between males and *LRRK2* G2385R carrier status (OR = 0.85, 95% CI: 0.70−1.02, *P*=0.08, Supplementary Figure 2).

#### 3.2.3. Disease Duration

There were seven studies that mentioned the difference in disease duration between *LRRK2* G2385R-carrier PD patients and *LRRK2* G2385R-noncarrier PD patients. The average disease duration in carriers of the *LRRK2* G2385R was slightly longer than that in noncarriers (MD = 0.47, 95% CI: 0.01−0.93, *P*=0.04, Supplementary Figure 3).

#### 3.2.4. Age at Onset

Twelve studies assessed the relationship between *LRRK2* G2385R status and age at onset (AAO) of PD patients. Four studies referred to AAO in early-onset PD patients. Another four studies were concerned with late-onset PD patients. No statistically significant differences were recorded between *LRRK2* G2385R-carrier PD group and *LRRK2* G2385R-noncarrier PD group (mean AAO: MD = −0.06, 95% CI: −0.15 to 0.03, *P*=0.19, Supplementary Figure 4A; AAO in early-onset PD: MD = −2.43, 95% CI: −0.55 to 5.42, *P*=0.11, Supplementary Figure 4B; AAO in late-onset PD: MD = −1.54, 95% CI: −3.38 to 0.30, *P*=0.10, Supplementary Figure 4C).

#### 3.2.5. Initial Symptoms

Four common initial symptoms, including tremor, rigidity, bradykinesia, and postural instability, were compared between *LRRK2* G2385R-carrier PD group and *LRRK2* G2385R-noncarrier PD group. No significant differences were observed (tremor: OR = 1.05, 95% CI: 0.76−1.45, *P*=0.76, Supplementary Figure 5A; rigidity: OR = 1.14, 95% CI: 0.70−1.86, *P*=0.60, Supplementary Figure 5B; bradykinesia: OR = 1.17, 95% CI: 0.76−1.79, *P*=0.48, Supplementary Figure 5C; postural instability: OR = 0.97, 95% CI: 0.39−2.39, *P*=0.94, Supplementary Figure 5D).

#### 3.2.6. Disease Severity

The H-Y and UPDRS-III were commonly used to evaluate disease severity. Six studies involved H-Y analysis, and five studies involved UPDRS-III analysis in *LRRK2* G2385R-carrier PD and *LRRK2* G2385R-noncarrier PD patients. No significant differences were discovered (H-Y: MD = −0.09, 95% CI: −0.20 to 0.01, *P*=0.07, Supplementary Figure 6A; UPDRS-III: MD = −1.35, 95% CI: −2.82 to 0.11, *P*=0.07, Supplementary Figure 6B).

#### 3.2.7. Motor Symptoms

Tremor, rigidity, bradykinesia, PIGD are the main motor manifestations of PD patients. Four studies assessed the relationship between *LRRK2* G2385R status and the tremor phenotype of PD patients. Three studies focused on the PIGD phenotype, and two studies analyzed rigidity and bradykinesia phenotype. The meta-analysis results of these studies showed that there were no significant differences in the above motor phenotypes in the *LRRK2* G2385R-carrier PD group compared to *LRRK2* G2385R-noncarrier PD group (tremor: OR = 0.88, 95% CI: 0.40−1.91, *P*=0.74, Supplementary Figure 7A; rigidity: OR = 1.59, 95% CI: 0.48−5.27, *P*=0.45, Supplementary Figure 7B; bradykinesia: OR = 0.43, 95% CI: 0.15−1.24, *P*=0.12, Supplementary Figure 7C; PIGD: OR = 1.24, 95% CI: 0.48−3.23, *P*=0.66, Supplementary Figure 7D).

#### 3.2.8. Nonmotor Symptoms

Only MMSE scores and the proportion of patients with depression were assessed owing to incomplete data about other nonmotor symptoms. Three studies included an MMSE score analysis in both groups and showed a significantly higher MMSE score in the *LRRK2* G2385R-carrier PD group than in the *LRRK2* G2385R-noncarrier PD group (MD = 1.02, 95% CI: 0.43–1.62, *P*=0.0007, Supplementary Figure 8A). Two studies included a depression analysis, but there was no significant association between depression and *LRRK2* G2385R status (OR = 1.46, 95% CI: 0.90−2.37, *P*=0.13, Supplementary Figure 8B).

#### 3.2.9. Levodopa Therapy and Related Complications

There were some studies that mentioned the LED and related complications, including motor fluctuation and dyskinesia. The meta-analysis results of these studies showed no significant differences for LED, motor fluctuation, or dyskinesia between the *LRRK2* G2385R-carrier PD group and the *LRRK2* G2385R-noncarrier PD group (LED: MD = 0.14, 95% CI: −0.06 to 0.33, *P*=0.17, Supplementary Figure 9A; motor fluctuation: OR = 1.73, 95% CI: 0.99−3.01, *P*=0.05, Supplementary Figure 9B; dyskinesia: OR = 1.26, 95% CI: 0.63−2.54, *P*=0.52, Supplementary Figure 9C).

Taken together, the *LRRK2* G2385R-carrier PD group more often presented a family history of PD and had a longer disease duration and a higher MMSE score than the *LRRK2* G2385R-noncarrier PD group. However, *LRRK2* G2385R carriers showed no significant differences in sex distribution, AAO, initial symptoms, motor symptoms and grade of severity, depression, or LED and levodopa-related complications compared to *LRRK2* G2385R noncarriers. The results of the meta-analysis are summarized in Table 2.

### 3.3. Sensitivity Analysis and Publication Bias

A sensitivity analysis was made in comparison with significant heterogeneity among the studies. When the heterogeneity among the studies was significant, we performed sensitivity analyses by excluding the relatively low-quality studies; the same results were obtained. Therefore, we can conclude that the sensitivity is low and that the results are reliable. As there were fewer than 10 high-quality studies in most comparisons in our meta-analysis, an accurate publication bias assessment could not be performed. However, we completed a funnel plot to estimate publication bias. There were no asymmetries in the funnel plot and no significant publication biases of the meta-analysis.

## 4. Discussion

In this meta-analysis, we extensively analyzed the relationship between *LRRK2* G2385R carrier status and clinical manifestations of PD in an Asian population. The *LRRK2* G2385R-carrier PD group significantly more often had a family history of PD than did the *LRRK2* G2385R-noncarrier PD group. The average disease duration in the *LRRK2* G2385R-carrier PD group was slightly longer than that in the *LRRK2* G2385R-noncarrier PD group. The mean MMSE score was significantly lower in the *LRRK2* G2385R-noncarrier PD group than in the *LRRK2* G2385R-carrier PD group.

Additionally, we observed that there were no significant differences in most of the clinical characteristics, including sex distribution, AAO, initial symptoms, motor symptoms and grade of severity, depression, and LED and levodopa-related complications between *LRRK2* G2385R-carrier and *LRRK2* G2385R-noncarrier PD patients. These results indicated that PD patients carrying a *LRRK2* G2385R mutation is associated with a significantly overlapping phenotype when compared with idiopathic PD. This finding is in line with those of previous studies reporting that G2019S carrier patients exhibit clinical features quite similar to those of noncarriers, with some mild differences [24–26]. One possible reason for this was that a broad spectrum of clinical characteristics may be caused by gene-gene interaction or gene-environment interactions, affecting the genotype and progression of the disease.

Nonmotor symptoms in PD are quite common and contribute to the patient's disability [27]. Associations between the genotype and nonmotor symptoms in PD patients have attracted research attention in recent years. To date, a few studies have focused mainly on several specific nonmotor symptoms, rather than on the general nonmotor symptom profile. Therefore, we failed to find sufficient data to assess the nonmotor symptoms of PD patients in the context of *LRRK2* G2385R status. For MMSE scores, given that only three published papers were included in the meta-analysis, a publication bias is likely to be present. A detailed clinical characterization of PD patients carrying *LRRK2* variants is warranted.

The NOS scores of all included publications were rated from 6 points to 9 points, providing a reliable bottom for the current analysis. No statistically significant publication bias or heterogeneity between different studies was detected in our meta-analysis. However, several limitations may have influenced the precision of our results. First, given that the number of included studies was limited, publication bias could potentially occur, even though we used various searching approaches and statistical analyses to minimize the publication bias. Second, although this meta-analysis included all the published case-control cohorts, some unpublished results may have been neglected.

## 5. Conclusion

In conclusion, our study suggested that most of the clinical characteristics of PD patients with the *LRRK2* G2385R mutation were similar to those of *LRRK2* G2385R noncarriers among Asian individuals, except for a more common family history, relatively longer disease duration, and higher MMSE scores in the *LRRK2* G2385R carriers. These findings strengthen our understanding of the clinical and genetic heterogeneity of PD and may have implications for diagnosis and therapy of PD. However, further larger samples and multicenter cooperative studies in different populations are warranted to clarify the relevance between the *LRRK2* G2385R genotype and clinical phenotype of PD.

## Figures and Tables

**Table 1:** Characteristics of included publication.

Article	Country	*LRRK2* G2385R	Number	Family history	Sex (male)	Disease duration	AAO (*n*)	AAO (EOPD)	AAO (LOPD) (*n*)	Initial symptom				H-Y	UPDRS-III	Motor symptom				Nonmotor symptoms		LED (mg)	Motor fluctuation	Dyskinesia	NOS score
										Tremor	Bradykinesia	PI	Rigidity			Tremor	Rigidity	Bradykinesia	PIGD	MMSE	Depression				
An et al. [5]	China	Carriers	71	—	40	—	52.75 ± 9.84	42.96 ± 7.23	58.40 ± 5.93	37	—	—	—	2.16 ± 0.91	—	—	—	—	—	—	—	—	—	—	8
		noncarriers	529	—	314	—	56.32 ± 11.26	40.81 ± 7.35	61.07 ± 7.27	263	—	—	—	2.33 ± 0.98	—	—	—	—	—	—	—	—	—	—	

Cai et al. [10]	China	Carriers	49	—	28	5.18 ± 2.58	56.8 ± 10.7	41.6 ± 5.91	61.3 ± 7.04	34	24	0	18	2.47 ± 0.89	21.4 ± 8.78	—	—	—	—	—	—	—	—	—	8
		noncarriers	461	—	275	4.92 ± 3.36	58.6 ± 11.0	42.9 ± 5.5	63.2 ± 7.33	299	224	9	168	2.57 ± 0.93	22.1 ± 8.35	—	—	—	—	—	—	—	—	—	

Cao et al. [7]	China	Carriers	14	0	5	8.57 ± 8.71	57.1 ± 11.0	—	—	8	7	5	7	1.7 ± 0.4	19.6 ± 5.1	—	—	—	—	—	—	—	—	3	7
		noncarriers	221	13	128	5.52 ± 4.81	54.4 ± 13.4	—	—	134	102	64	83	2.0 ± 0.8	20.5 ± 7.3	—	—	—	—	—	—	—	—	57	

Cao et al. [16]	China	Carriers	25	—	16	—	—	—	—	—	—	—	—	2.2 ± 0.8	—	—	—	—	—	—	—	—	—	—	5
		noncarriers	43	—	28	—	—	—	—	—	—	—	—	2.4 ± 0.74	—	—	—	—	—	—	—	—	—	—	

Chan et al. [17]	Hong Kong	Carriers	—	—	—	—	—	42.7 ± 2.3 (3)	74.3 ± 4.9 (4)	—	—	—	—	—	—	—	—	—	—	—	—	—	—	—	6
	China	noncarriers	—	—	—	—	—	38.3 ± 6.6 (34)	69.2 ± 10.7 (44)	—	—	—	—	—	—	—	—	—	—	—	—	—	—	—	

Di Fonzo et al. [18]	Taiwan	Carriers	61	—	34	13.4 ± 6.2	53.2 ± 12.5	—	—	—	—	—	—	—	—	—	—	—	—	—	—	—	—	—	8
	China	noncarriers	547	—	328	11.2 ± 6.0	55.0 ± 11.9	—	—	—	—	—	—	—	—	—	—	—	—	—	—	—	—	—	

Farrer et al. [3]	Taiwan	Carriers	34	6	18	7.3 ± 5.4	54.9 ± 11.8	—	—	—	—	—	—	—	—	24	32	30	12	—	—	457.6 ± 246.6	12	13	8
	China	noncarriers	376	20	214	7.2 ± 4.6	58.0 ± 11.7	—		—	—	—	—	—	—	258	343	365	150	—	—	444.6 ± 270.9	122	117	

Funayama et al. [20]	Japan	Carriers	—	—	—	—	54.0 ± 10.6 (50)	42.5 ± 5.8 (17)	59.9 ± 7.0 (33)	—	—	—	—	—	—	—	—	—	—	—	—	—	—	—	7
		noncarriers	—	—	—	—	50.3 ± 14.9 (389)	37.1 ± 9.4 (180)	61.6 ± 7.8 (209)	—	—	—	—	—	—	—	—	—	—	—	—	—	—	—	

Fung et al. [22]	Taiwan	Carriers	27	—	—	—	61.5 ± 11.3	—	—	—	—	—	—	—	—	26	26	27	—	—	—	—	—	—	7
	China	noncarriers	278	—	—	—	61.9 ± 10.5		—	—	—	—	—	—	—	252	261	269	—	—	—	—	—	—	

Fu et al. [19]	China	Carriers	37	8	21	—	55 ± 11.7	—	—	21	11	1	3	—	—	—	—	—	—	—	—	—	—	—	7
		noncarriers	365	51	201	—	55.0 ± 11.0	—	—	220	81	16	26	—	—	—	—	—	—	—	—	—	—	—	

Gao et al. [7]	China	Carriers	36	2	21	4.1 ± 2.7	60.5 ± 10.4	—	—	—	—	—	—	—	—	16	—	—	16	27.2 ± 3.4	—	—	13	6	6
		noncarriers	139	10	73	3.8 ± 3.2	58.2 ± 8.8	—	—	—	—	—	—	—	—	55	—	—	73	25.3 ± 4.0	—	—	15	6	

Hong et al. [21]	Korean	Carriers	23	—	7	3.8 ± 3.1	—	—	—	—	—	—	—	—	19.8 ± 11.2	—	—	—	—	25.6 ± 4.4	5	—	—	—	7
		noncarriers	276	—	136	4.2 ± 3.3	—	—	—	—	—	—	—	—	23.6 ± 8.7	—	—	—	—	25.0 ± 4.0	56	—	—	—	

Kim et al. [9]	Korean	Carriers	82	—	35		55.3 ± 11.5	—	—	—	—	—	—	—	—	—	—	—	—	—	—	—	—	—	9
		noncarriers	841	—	373		54.7 ± 10.8	—	—	—	—	—	—	—	—	—	—	—	—	—	—	—	—	—	

Sun et al. [6]	China	Carriers	76	—	37	6.57 ± 4.21	55.57 ± 8.78	—	—	—	—	—	—	2.15 ± 0.83	24.55 ± 12.08	29	—	—	43	27.78 ± 2.68	27	553.67 ± 329.84	27	4	7
		noncarriers	225	—	125	5.73 ± 3.55	57.05 ± 8.92	—	—	—	—	—	—	2.08 ± 0.81	26.52 ± 12.64	140	—	—	69	26.96 ± 2.92	58	467.71 ± 286.34	50	8	

Tan et al. [8]	Singapore	Carriers	33	5	16	—	59.0. ± 11.0	—	—	—	—	—	—	—	26.0 ± 11.0	—	—	—	—	—	—	467.0 ± 275.0	—	7	5
		noncarriers	29	0	18	—	59.0 ± 10.0		—	—	—	—	—	—	27.0 ± 11.0	—	—	—	—	—	—	537.0 ± 287.0	—	11	

Zhou et al. [23]	China	Carriers	26	—	—	—	—	—	—	—	—	—	—	1.82 ± 0.58	—	—	—	—	—	—	—	—	—	—	7
		noncarriers	176	—	—	—	—	—	—	—	—	—	—	1.76 ± 0.76	—	—	—	—	—	—	—	—	—	—	

**Table 2:** Summarized results of the meta-analysis.

Subjects	Effect model	MD/OR (95% CI)	*P* value	Study heterogeneity			
				Chi^2^	df	*I* ^2^ (%)	*P* value
Family history	Fixed	1.98 [1.16–3.39]	0.01	5.42	4	26	0.25
Gender (male)	Fixed	0.85 [0.70–1.02]	0.08	6.09	11	0	0.87
Disease duration	Fixed	0.47 [0.01–0.93]	0.04	8.16	6	27	0.23
AAO	Fixed	−0.06 [−0.15 to 0.03]	0.19	16.32	11	33	0.13
AAO (early-onset)	Random	2.43 [−0.55 to 5.42]	0.11	19.47	3	85	0.0002
AAO (late-onset)	Random	−1.54 [−3.38 to 0.30]	0.10	6.67	3	55	0.08
Initial symptoms—tremor	Fixed	1.05 [0.76–1.45]	0.76	0.70	3	0	0.87
Initial symptoms—rigidity	Fixed	1.14 [0.70–1.86]	0.60	0.61	2	0	0.74
Initial symptoms—bradykinesia	Fixed	1.17 [0.76–1.79]	0.48	0.61	2	0	0.74
Initial symptoms—PI	Fixed	0.97 [0.39–2.39]	0.94	0.78	2	0	0.68
H-Y	Fixed	−0.09 [−0.20 to 0.01]	0.07	7.39	5	32	0.19
UPDRS-III	Fixed	−1.35 [−2.82 to 0.11]	0.07	1.56	4	0	0.82
Motor symptoms—tremor	Random	0.88 [0.40–1.91]	0.74	10.41	3	71	0.02
Motor symptoms—rigidity	Fixed	1.59 [0.48–5.27]	0.45	0.01	1	0	0.94
Motor symptoms—bradykinesia	Fixed	0.43 [0.15–1.24]	0.12	1.90	1	47	0.17
Motor symptoms—PIGD	Random	1.24 [0.48–3.23]	0.66	12.47	2	84	0.002
MMSE	Fixed	1.02 [0.43–1.62]	0.0007	2.27	2	12	0.32
Depression	Fixed	1.46 [0.90–2.37]	0.13	0.39	1	0	0.53
LED	Fixed	0.14 [−0.06 to 0.33]	0.17	3.78	2	47	0.15
Motor fluctuation	Random	1.73 [0.99–3.01]	0.05	7.52	2	73	0.02
Dyskinesia	Random	1.26 [0.63–2.54]	0.52	8.26	4	52	0.08

## Data Availability

The data used to support the findings of this study are available from the corresponding author upon request.

## Supplementary Materials

Supplementary Materials Supplementary Figure 1: forest plot of family history in *LRRK2* G2385R+PD and *LRRK2* G2385R-PD. Supplementary Figure 2: forest plot of gender distribution (male) in *LRRK2* G2385R+PD and *LRRK2* G2385R-PD. Supplementary Figure 3: forest plot of disease duration in *LRRK2* G2385R+PD and *LRRK2* G2385R-PD. Supplementary Figure 4: forest plot of age at onset in *LRRK2* G2385R+PD and *LRRK2* G2385R-PD—(A) mean AAO, (B) AAO in early-onset PD, and (C) AAO in late-onset PD. Supplementary Figure 5: forest plot of initial symptoms in *LRRK2* G2385R+PD and *LRRK2* G2385R-PD—(A) tremor, (B) rigidity, (C) bradykinesia, and (D) postural instability. Supplementary Figure 6: forest plot of motor symptoms severity in *LRRK2* G2385R+PD and *LRRK2* G2385R-PD—(A) H-Y and (B) UPDRS. Supplementary Figure 7: forest plot of motor symptoms in *LRRK2* G2385R+PD and *LRRK2* G2385R-PD—(A) tremor, (B) rigidity, (C) bradykinesia, and (D) PIGD. Supplementary Figure 8: forest plot of nonmotor symptoms in *LRRK2* G2385R+PD and *LRRK2* G2385R-PD—(A) MMSE and (B) depression. Supplementary Figure 9: forest plot of levodopa therapy and related complications in *LRRK2* G2385R+PD and *LRRK2* G2385R-PD—(A) levodopa-equivalent dose, (B) motor fluctuation, and (C) dyskinesia.

## References

[1] Tolleson C. M., Fang J. Y. (2013). Advances in the mechanisms of Parkinson’s disease. *Discovery Medicine*.

[2] Healy D. G., Falchi M., O’Sullivan S. S. (2008). Phenotype, genotype, and worldwide genetic penetrance of LRRK2-associated Parkinson’s disease: a case-control study. *Lancet Neurology*.

[3] Farrer M. J., Stone J. T., Lin C. H. (2007). Lrrk2 G2385R is an ancestral risk factor for Parkinson’s disease in Asia. *Parkinsonism and Related Disorders*.

[4] Cao L., Zhang T., Xiao Q. (2007). The prevalence of LRRK2 Gly2385Arg variant in Chinese Han population with Parkinson’s disease. *Movement Disorders*.

[5] An X. K., Peng R., Li T. (2008). LRRK2 Gly2385Arg variant is a risk factor of Parkinson’s disease among Han-Chinese from mainland China. *European Journal of Neurology*.

[6] Sun Q., Wang T., Jiang T. F. (2016). Effect of a leucine-rich repeat kinase 2 variant on motor and non-motor symptoms in Chinese Parkinson’s disease patients. *Aging and Disease*.

[7] Gao C., Pang H., Luo X. G., Ren Y., Shang H., He Z. Y. (2013). LRRK2 G2385R variant carriers of female Parkinson’s disease are more susceptible to motor fluctuation. *Journal of Neurology*.

[8] Tan E. K., Fook-Chong S., Yi Z. (2007). Comparing LRRK2 Gly2385Arg carriers with noncarriers. *Movement Disorders*.

[9] Kim J. M., Lee J. Y., Kim H. J. (2010). The LRRK2 G2385R variant is a risk factor for sporadic Parkinson’s disease in the Korean population. *Parkinsonism and Related Disorders*.

[10] Cai J., Lin Y., Chen W. (2013). Association between G2385R and R1628P polymorphism of LRRK2 gene and sporadic Parkinson’s disease in a Han-Chinese population in south-eastern China. *Neurological Sciences*.

[11] Stroup D. F., Berlin J. A., Morton S. C. (2000). Meta-analysis of observational studies in epidemiology: a proposal for reporting. Meta-analysis of Observational Studies in Epidemiology (MOOSE) group. *JAMA*.

[12] Liberati A., Altman D. G., Tetzlaff J. (2009). The PRISMA statement for reporting systematic reviews and meta-analyses of studies that evaluate health care interventions: explanation and elaboration. *Journal of Clinical Epidemiology*.

[13] Fan X., Lin T., Xu K. (2012). Laparoendoscopic single-site nephrectomy compared with conventional laparoscopic nephrectomy: a systematic review and meta-analysis of comparative studies. *European Urology*.

[14] Wells G. A. S. B., O’Connell D., Peterson J., Welch V., Losos M. T. P. (July 2015). *The Newcastle-Ottawa Scale (NOS) for Assessing the Quality of Nonrandomised Studies in Meta-Analyses*.

[15] Higgins J. P. T., Green S. (2008). *Cochrane Handbook for Systematic Reviews of Interventions. Cochrane Collaboration*.

[16] Cao M., Gu Z. Q., Li Y. (2016). Olfactory dysfunction in Parkinson’s disease patients with the LRRK2 G2385R variant. *Neuroscience Bulletin*.

[17] Chan D. K., Ng P. W., Mok V. (2008). LRRK2 Gly2385Arg mutation and clinical features in a Chinese population with early-onset Parkinson’s disease compared to late-onset patients. *Journal of Neural Transmission*.

[18] Di Fonzo A., Wu-Chou Y. H., Lu C. S. (2006). A common missense variant in the LRRK2 gene, Gly2385Arg, associated with Parkinson’s disease risk in Taiwan. *Neurogenetics*.

[19] Fu X., Zheng Y., Hong H. (2013). LRRK2 G2385R and LRRK2 R1628P increase risk of Parkinson’s disease in a Han Chinese population from Southern Mainland China. *Parkinsonism and Related Disorders*.

[20] Funayama M., Li Y., Tomiyama H. (2007). Leucine-rich repeat kinase 2 G2385R variant is a risk factor for Parkinson disease in Asian population. *Neuroreport*.

[21] Hong J. H., Kim Y. K., Park J. S. (2017). Lack of association between LRRK2 G2385R and cognitive dysfunction in Korean patients with Parkinson’s disease. *Journal of Clinical Neuroscience*.

[22] Fung H. C., Chen C. M., Hardy J., Singleton A. B., Wu Y. R. (2006). A common genetic factor for Parkinson disease in ethnic Chinese population in Taiwan. *BMC Neurology*.

[23] Zhou Y., Luo X., Li F. (2012). Association of Parkinson’s disease with six single nucleotide polymorphisms located in four PARK genes in the northern Han Chinese population. *Journal of Clinical Neuroscience*.

[24] Bouhouche A., Tibar H., Ben El Haj R. (2017). LRRK2 G2019S mutation: prevalence and clinical features in Moroccans with Parkinson’s disease. *Parkinson’s Disease*.

[25] Alcalay R. N., Mirelman A., Saunders-Pullman R. (2013). Parkinson disease phenotype in Ashkenazi Jews with and without LRRK2 G2019S mutations. *Movement Disorders*.

[26] Li D. W., Gu Z., Wang C. (2015). Non-motor symptoms in Chinese Parkinson’s disease patients with and without LRRK2 G2385R and R1628P variants. *Journal of Neural Transmission*.

[27] Bostantjopoulou S., Katsarou Z., Karakasis C., Peitsidou E., Milioni D., Rossopoulos N. (2013). Evaluation of non-motor symptoms in Parkinson’s Disease: an underestimated necessity. *Hippokratia*.

