# Supplementary material for: The Association between LRRK2 G2385R and Phenotype of Parkinson's Disease in Asian Population: A Meta-Analysis of Comparative Studies
Source: Parkinsons Dis. 2018 Jul 10;2018:3418306. doi: 10.1155/2018/3418306 (PMC6079378; doi:10.1155/2018/3418306)
Supplement: Supplementary Materials — Supplementary Figure 1: forest plot of family history in LRRK2 G2385R+PD and LRRK2 G2385R-PD. Supplementary Figure 2: forest plot of gender distribution (male) in LRRK2 G2385R+PD and LRRK2 G2385R-PD. Supplementary Figure 3: forest plot of disease duration in LRRK2 G2385R+PD and LRRK2 G2385R-PD. Supplementary Figure 4: forest plot of age at onset in LRRK2 G2385R+PD and LRRK2 G2385R-PD—(A) mean AAO, (B) AAO in early-onset PD, and (C) AAO in late-onset PD. Supplementary Figure 5: forest plot of initial symptoms in LRRK2 G2385R+PD and LRRK2 G2385R-PD—(A) tremor, (B) rigidity, (C) bradykinesia, and (D) postural instability. Supplementary Figure 6: forest plot of motor symptoms severity in LRRK2 G2385R+PD and LRRK2 G2385R-PD—(A) H-Y and (B) UPDRS. Supplementary Figure 7: forest plot of motor symptoms in LRRK2 G2385R+PD and LRRK2 G2385R-PD—(A) tremor, (B) rigidity, (C) bradykinesia, and (D) PIGD. Supplementary Figure 8: forest plot of nonmotor symptoms in LRRK2 G2385R+PD and LRRK2 G2385R-PD—(A) MMSE and (B) depression. Supplementary Figure 9: forest plot of levodopa therapy and related complications in LRRK2 G2385R+PD and LRRK2 G2385R-PD—(A) levodopa-equivalent dose, (B) motor fluctuation, and (C) dyskinesia. [file 3418306.f1.docx]

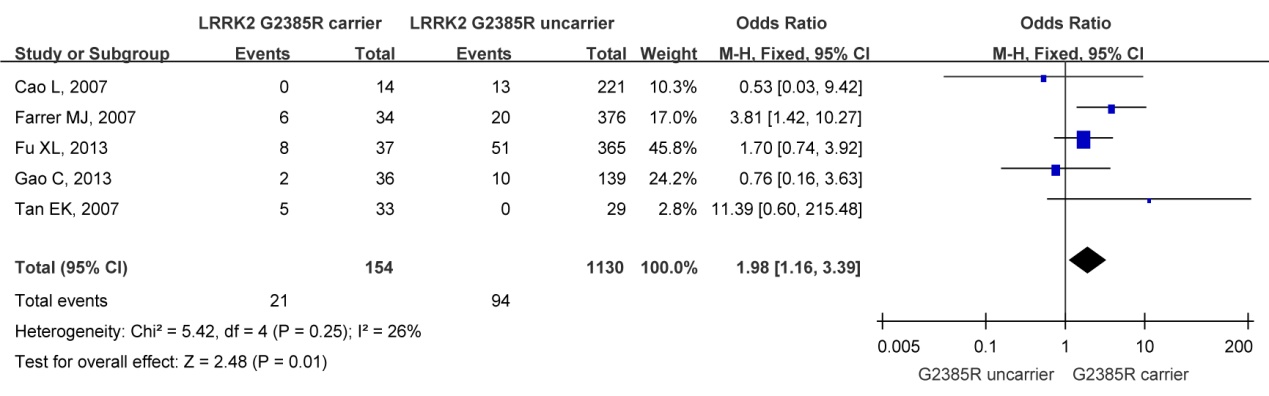


**Supplementary FIGURE 1:** Forest plot of family history in LRRK2 G2385R+PD and LRRK2 G2385R-PD.


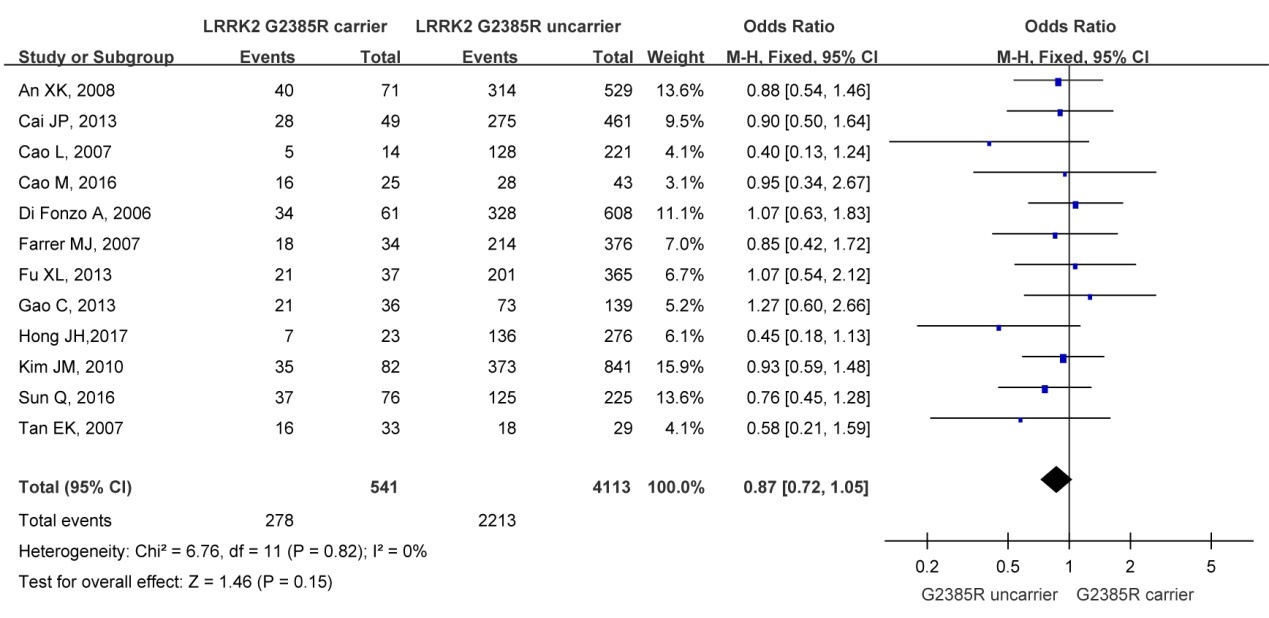


**Supplementary FIGURE 2:** Forest plot of gender distribution (male) in LRRK2 G2385R+PD and LRRK2 G2385R-PD.


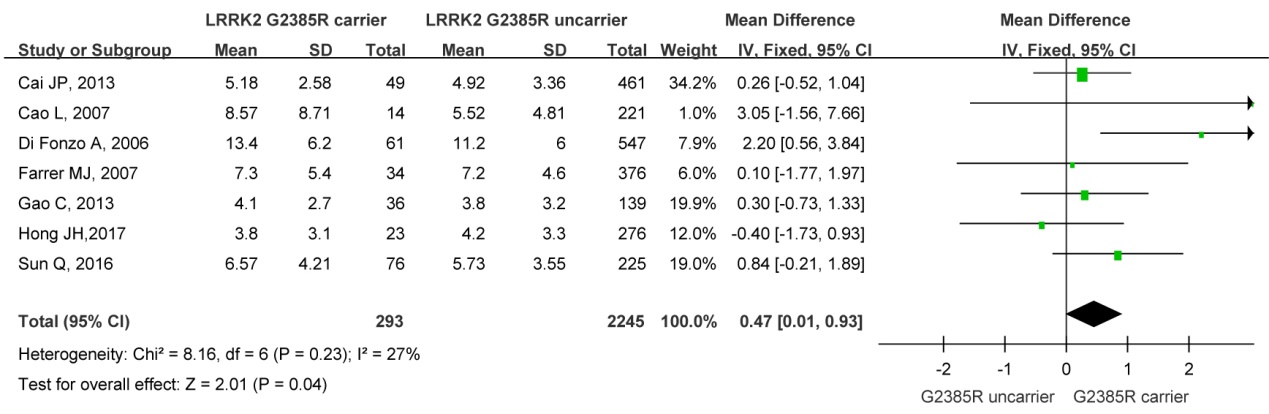


**Supplementary FIGURE 3.** Forest plot of disease duration in LRRK2 G2385R+PD and LRRK2 G2385R-PD.


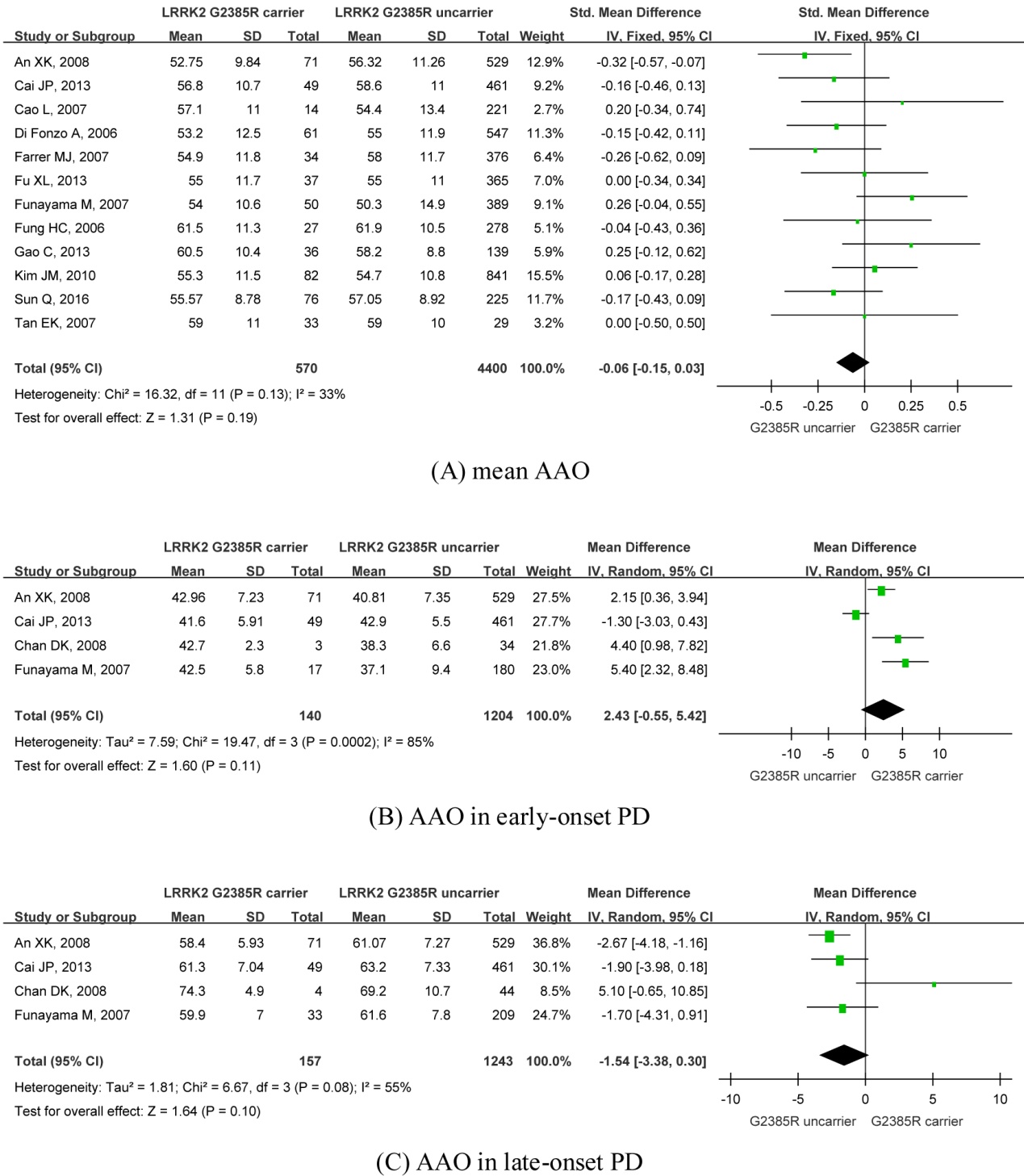


**Supplementary FIGURE** **4:** Forest plot of age at onset in LRRK2 G2385R+PD and LRRK2 G2385R-PD. (A) mean AAO. (B) AAO in early-onset PD. (C) AAO in late-onset PD.


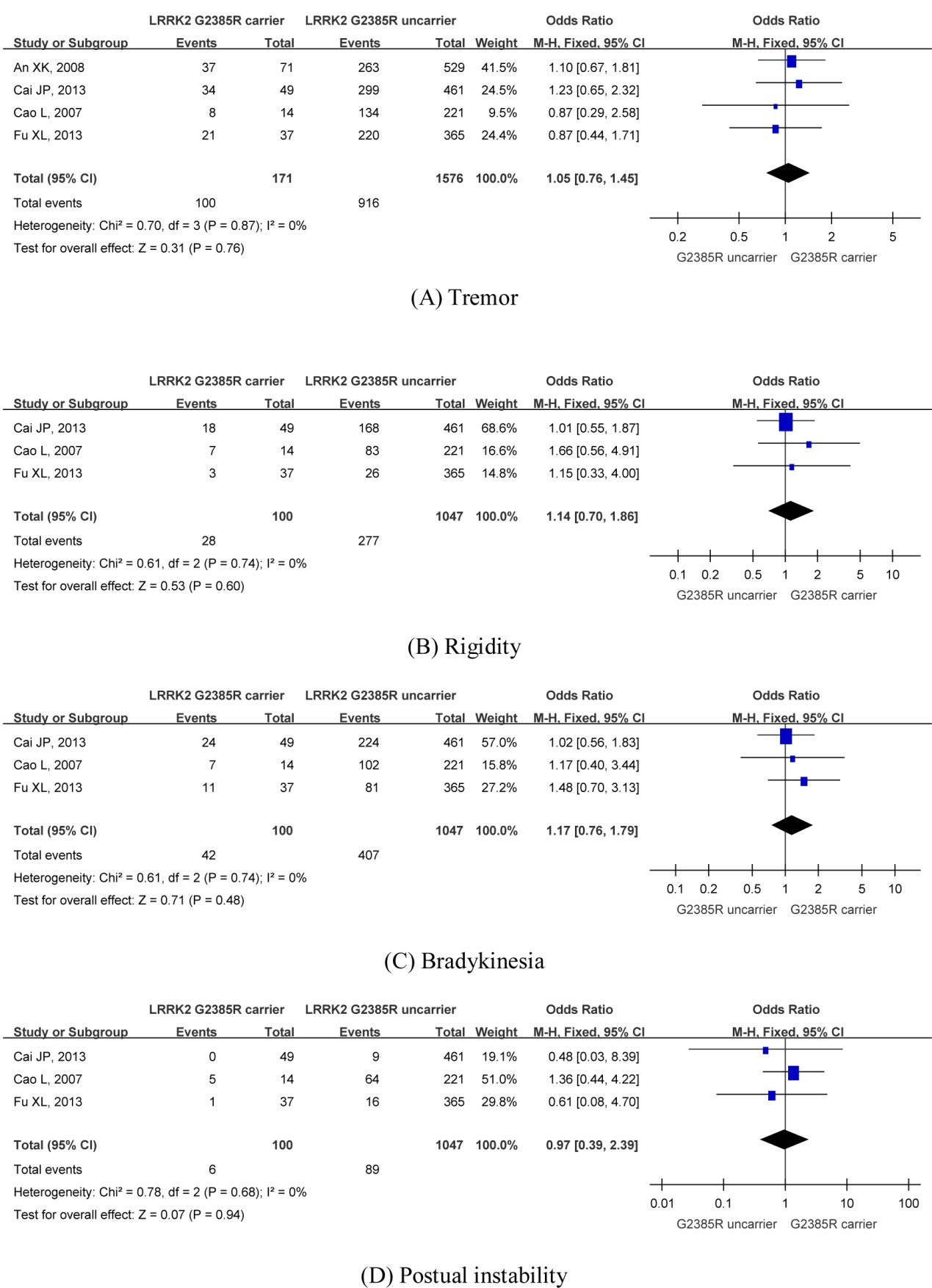


**Supplementary FIGURE 5:** Forest plot of initial symptoms in LRRK2 G2385R+PD and LRRK2 G2385R-PD. (A) Tremor. (B) Rigidity. (C) Bradykinesia. (D) Postual instability.


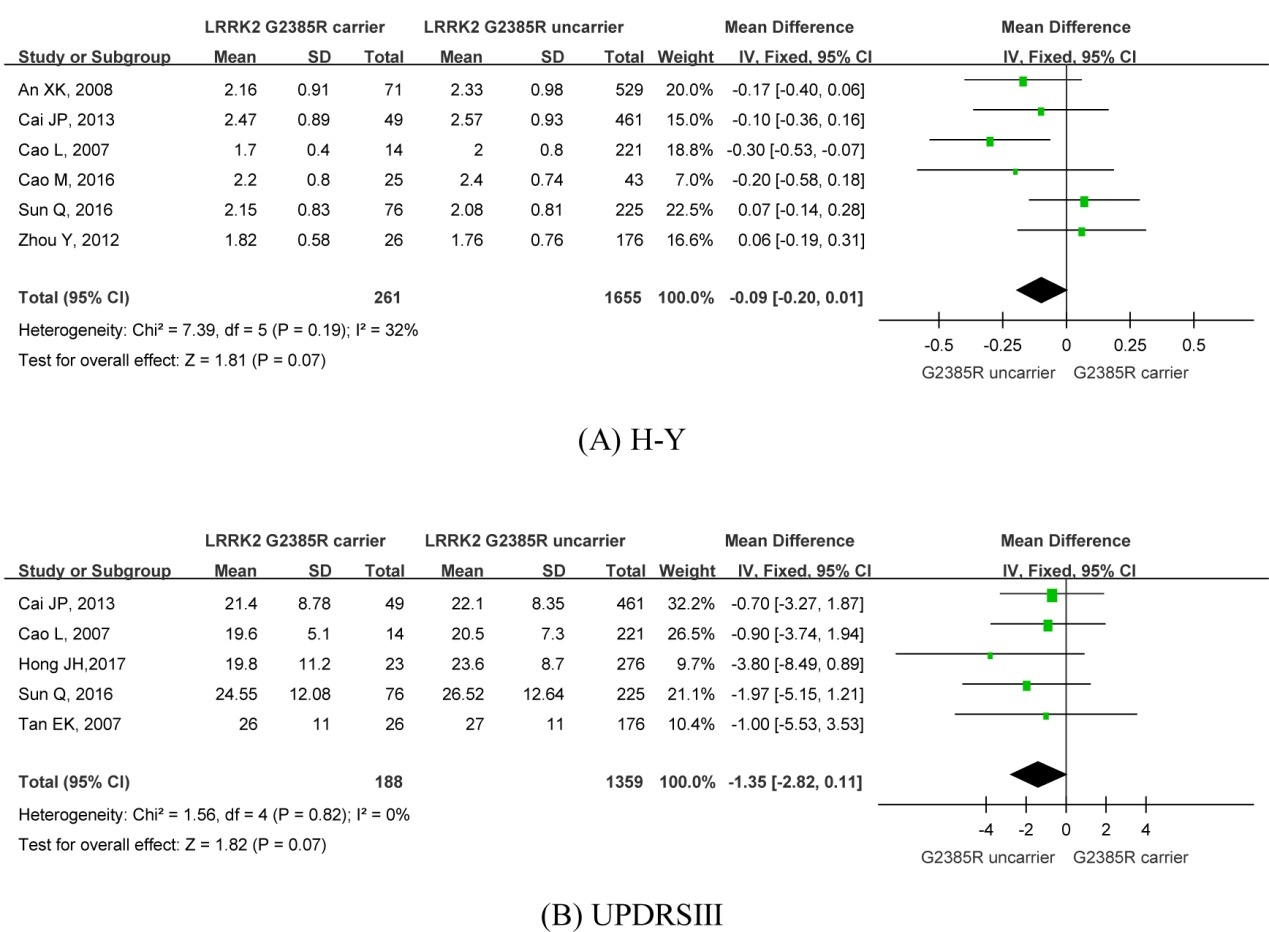


**Supplementary FIGURE** **6:** Forest plot of motor symptoms severity in LRRK2 G2385R+PD and LRRK2 G2385R-PD. (A) H-Y. (B) UPDRSⅢ.


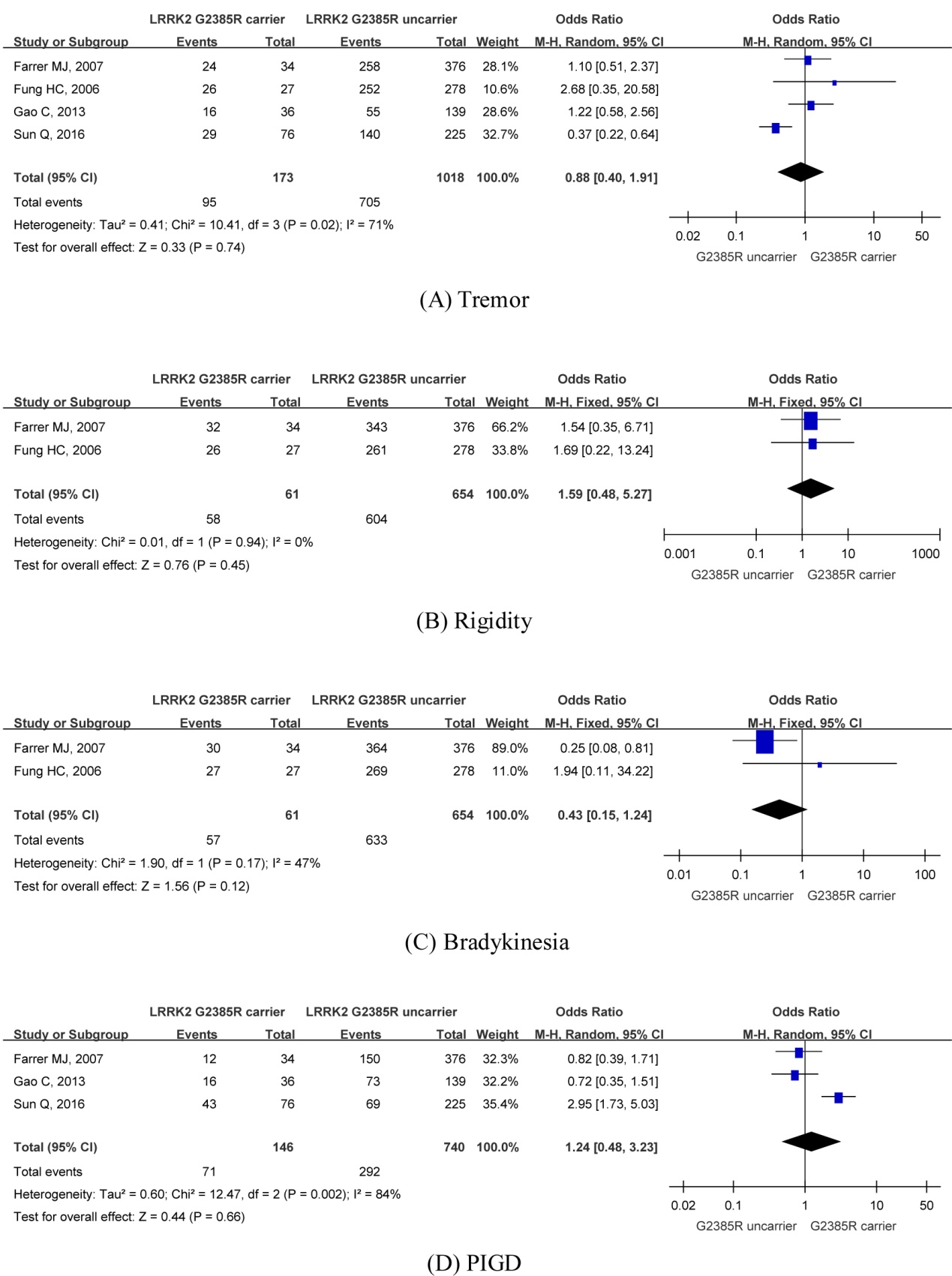


**Supplementary FIGURE** **7:** Forest plot of motor symptoms in LRRK2 G2385R+PD and LRRK2 G2385R-PD. (A) Tremor. (B) Rigidity. (C) Bradykinesia. (D) PIGD.

**
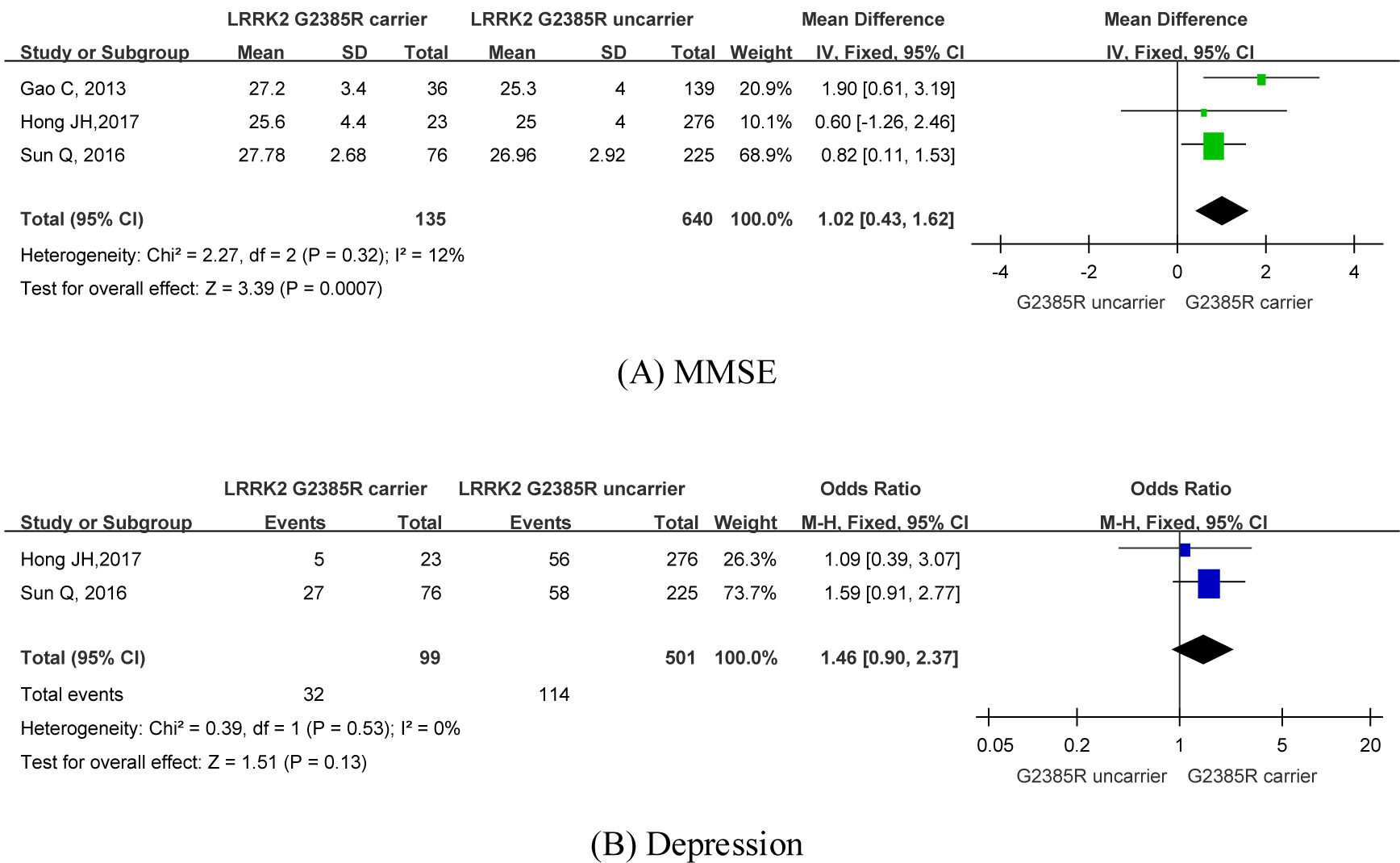
**

**Supplementary FIGURE** **8:** Forest plot of non-motor symptoms in LRRK2 G2385R+PD and LRRK2 G2385R-PD. (A) MMSE. (B) Depression.


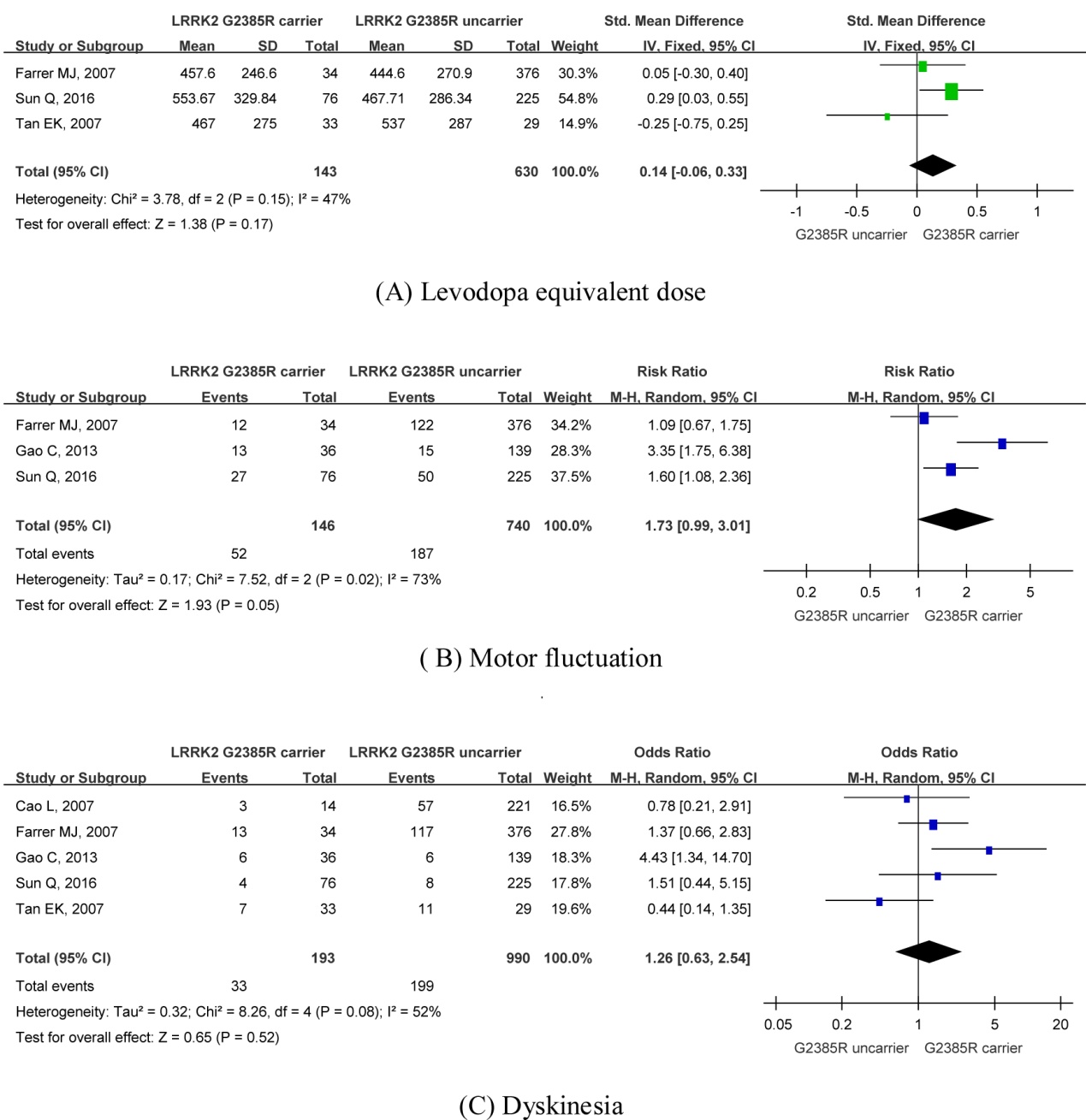


**Supplementary FIGURE** **9****:** Forest plot of levodopa therapy and related complications in LRRK2 G2385R+PD and LRRK2 G2385R-PD. (A) Levodopa equivalent dose. ( B) Motor fluctuation.. (C) Dyskinesia.
